# Green synthesis, crystal structure, and antifungal activities of (*E*)-4-arylidene-5-oxotetrahydrofuran

**DOI:** 10.3389/fchem.2022.997095

**Published:** 2022-09-09

**Authors:** Yun Dong, Ling-Qi Kong, Qin-Hua Chen, Bin Li, Xiao-Hua Zeng, Li-Na Ke, Hong-Mei Wang

**Affiliations:** ^1^ Sinopharm Dongfeng General Hospital, Hubei University of Medicine, Shiyan, China; ^2^ Hubei Key Laboratory of Wudang Local Chinese Medicine Research, School of Pharmaceutical Sciences, Hubei University of Medicine, Shiyan, China; ^3^ Shenzhen Baoan Authentic TCM Therapy Hospital, Shenzhen, China

**Keywords:** (E)-4-arylidene-5-oxotetrahydrofuran, green synthesis, ionic liquid, microwave-assisted one-pot, antifungal activity

## Abstract

A series of γ-lactone derivatives (*E*)-4-arylidene-5-oxotetrahydrofuran derivatives were synthesized *via* a tandem Passerini 3CC/S_N_ cyclization microwave-assisted one-pot method efficiently starting from Baylis Hillman acids, aryl glyoxals and isocyanides, and using ionic liquid as reaction medium. The products were characterized by hydrogen nuclear magnetic resonance spectroscopy (^1^H-NMR), carbon nuclear magnetic resonance spectroscopy (^13^C-NMR). Single crystal X-ray analysis of the compound RPDFB clearly confirmed its assigned chemical structures. Meanwhile, the effects of four compounds (RPDFB, RPDFC, RPDFI, RPDFJ) on the growth inhibition activity of *Gibberella zeae* were detected, and found that the compound RPDFB has significant growth inhibition activity to *Gibberella zeae*.

## 1 Introduction

The synthesis of heterocyclic compounds plays an important role in organic synthesis, because heterocyclic compounds have a large number and are widely used in pesticide, medicine and industry (El-Sayed and Althagafi, 2016). Among heterocyclic compounds, especially those containing pyrrole ring, pyridine ring, furan ring and β-lactam ring have attracted the attention of organic synthesis scientists because of their high efficiency, low toxicity and broad spectrum of physiological activities (Butin et al., 2010; Hafez and El-Gazzar, 2020; Shiri, 2021; Zhang et al., 2022). At present, numerous anti-inflammatory, antitumor, antiviral, antibacterial, insecticide, and herbicide containing these heterocyclic rings have been developed by synthetic methods (Burriss et al., 2018; Bari et al., 2019; Jiang et al., 2021; Manaithiya et al., 2021). Therefore, the structural modification and synthesis method optimization of heterocyclic compounds are very important for more efficient and green synthesis of new agents.

The development prospect of pharmaceutical and pesticide products is to synthesize agents which are environmentally friendly, safe and harmless to human beings, with high selectivity and ultra-low dosage by using green synthesis process (Sharma et al., 2020; Zhang et al., 2020). Therefore, green synthesis method will also be a mandatory requirement of green pesticides. Following this concept, we applied the efficient multi-component tandem one-pot method to synthesize heterocyclic compounds with potential biological activity, such as pyrrole, pyridine, furan, and β-lactam.

**SCHEME 1:**
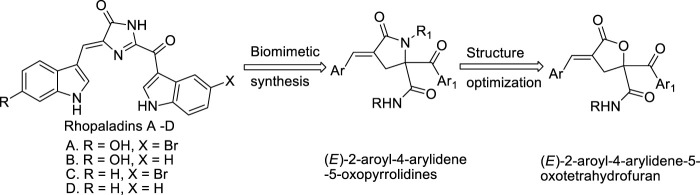
The strategy for the choice of (E)-2-aroyl-4-arylidene-5-oxotetrahydrofurans as targets.

**SCHEME 2:**
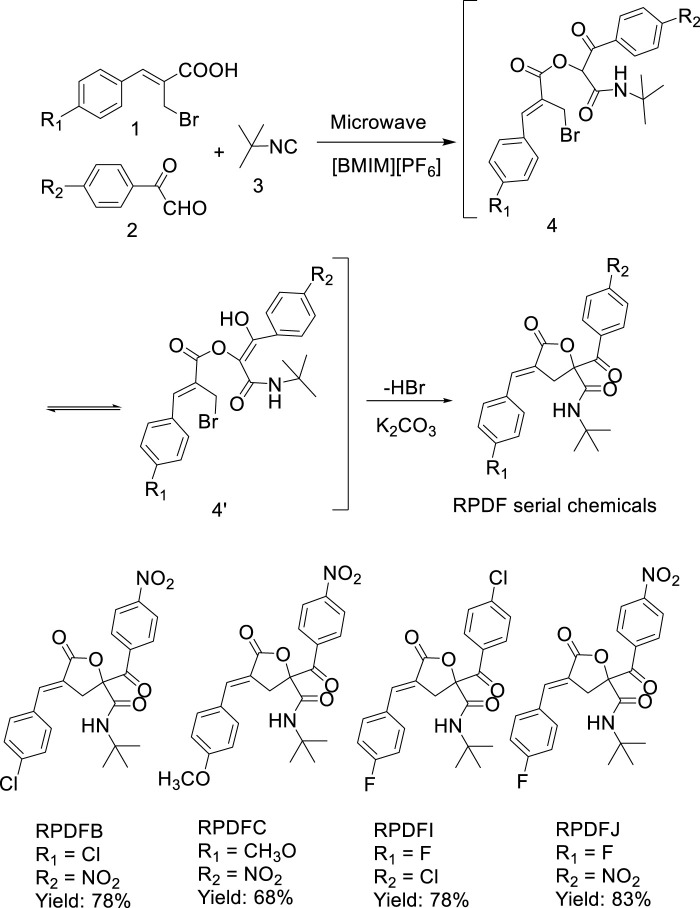
Synthesis of (*E*)-2-aroyl-4-arylidene-5-oxotetrahydrofuran derivatives.

Studies have shown that rhopaladins A-D alkaloids (Janosik et al., 2002; Méndez et al., 2018) (Scheme 1) present in marine cysts have significantly biological activities. We have previously synthesized several different series of rhopaladins’ analogs, such as (*E*)-2-aroyl-4-arylidene-5-oxopyrrolidines (Scheme 1), and (2*E*, 4*E*)-2-styryl-4-arylidene-5-oxopyrrolidines, *via* multi-component one-pot method starting from Baylis-Hillman acid, and found that these compounds showed good anti-proliferation and apoptosis-inducing effects on cervical cancer cells and liver cancer cells, respectively (Zeng et al., 2013; Zhu et al., 2020; Wang et al., 2021; Wang et al., 2021; Kong et al., 2021; Zhu et al., 2022). Baylis Hillman (B-H) reaction is a coupling reaction between active alkenes and electrophiles under catalyst, which can be used to synthesize compounds with polyfunctional groups under mild reaction conditions (Huang and Cui, 2018). Moreover, B-H reaction is also an important synthetic transition reaction in the formation of carbon-carbon bond, which can provide highly substituted allyl alcohol and amine in one step reaction (Nelson et al., 2018). γ-Lactone is a common chemical skeleton in many synthetic and natural products, mainly composed of five-member lactone heterocycles, which possess many biological properties and are key intermediates in the synthesis of major antiviral and anticancer nucleoside analogs (Julien et al., 2018). Therefore, by combining Passerini reaction with Baylis Hillman reaction, γ-lactone derivatives of (*E*)-4-arylidene-5-oxotetrahydrofuran derivatives (RPDF serial chemicals, which are other analogs of rhopaladins, Scheme 1) were synthesized *via* a tandem Passerini 3CC/S_N_ cyclization one pot method (Wang et al., 2021).

Herein, we optimized the synthesis method, using ionic liquid as a green medium and microwave radiation as a heating method to synthesize (*E*)-*N*-tert-butyl-4-(4-chlorobenzylidene)-2-(4-nitrobenzoyl)-5-oxotetrahydrofuran-2 -carboxamide (RPDFB) and other three new compounds (RPDFC, RPDFI, RPDFJ). Next, the single crystal of RPDFB was cultured and analyzed. Meanwhile, the effects of the four compounds (RPDFB, RPDFC, RPDFI, RPDFJ) on the growth inhibition activity of *Gibberella zeae* were detected.

## 2 Results and discussion

### 2.1 Chemistry

The realization of environmentally friendly organic synthesis has become an important topic of organic synthesis. Such as solvent-free reaction, or reaction with water as the medium and using room temperature ionic liquid as the reaction solvent (Welton, 1999). Ionic liquid has the advantages of low vapor pressure, non-flammable, good thermal stability, recyclable, compared with other organic solvents (Sowmiah et al., 2009; Çınar et al., 2016; Bystrzanowska et al., 2019). On the other hand, assisted by microwave radiation, the rate of organic reaction is also greatly improved, and simple operation, high yield, simple post-treatment characteristics (Santagada et al., 2009; Sharma et al., 2012). Meanwhile, study has shown that ionic liquid [BMIM][PF_6_] has a good role in the one-pot synthesis of tetrahydropyran ring in series Barbier-Prins reaction (Batista et al., 2019). Therefore, we tried to synthesize RPDFB RPDFC, RPDFI, RPDFJ in ionic liquid [BMIM][PF_6_] and microwave-assisted as the continuation of previous research.

The results showed that using Baylis-Hillmanic acid 1, aryl glyoxals 2, and isocyanide 3 as starting materials and ionic liquid [BMIM][PF_6_] as reaction medium, a series of γ-lactone derivatives (*E*)-2-aroyl-4-arylidene-5-oxotetrahydrofuran derivatives (see Scheme 2) were synthesized via a tandem Passerini 3CC/S_N_ cyclization microwave-assisted one-pot method efficiently, which provided a new green synthesis strategy for the synthesis of (*E*)-4-arylidene-5-oxotetrahydrofuran derivatives.

### 2.2 Crystal structure description of (E)-N-tert-butyl-4-(4-chlorobenzylidene)-2-(4-nitrobenzoyl)-5-oxotetrahydrofuran-2-carboxamide (RPDFB)

**FIGURE 1:**
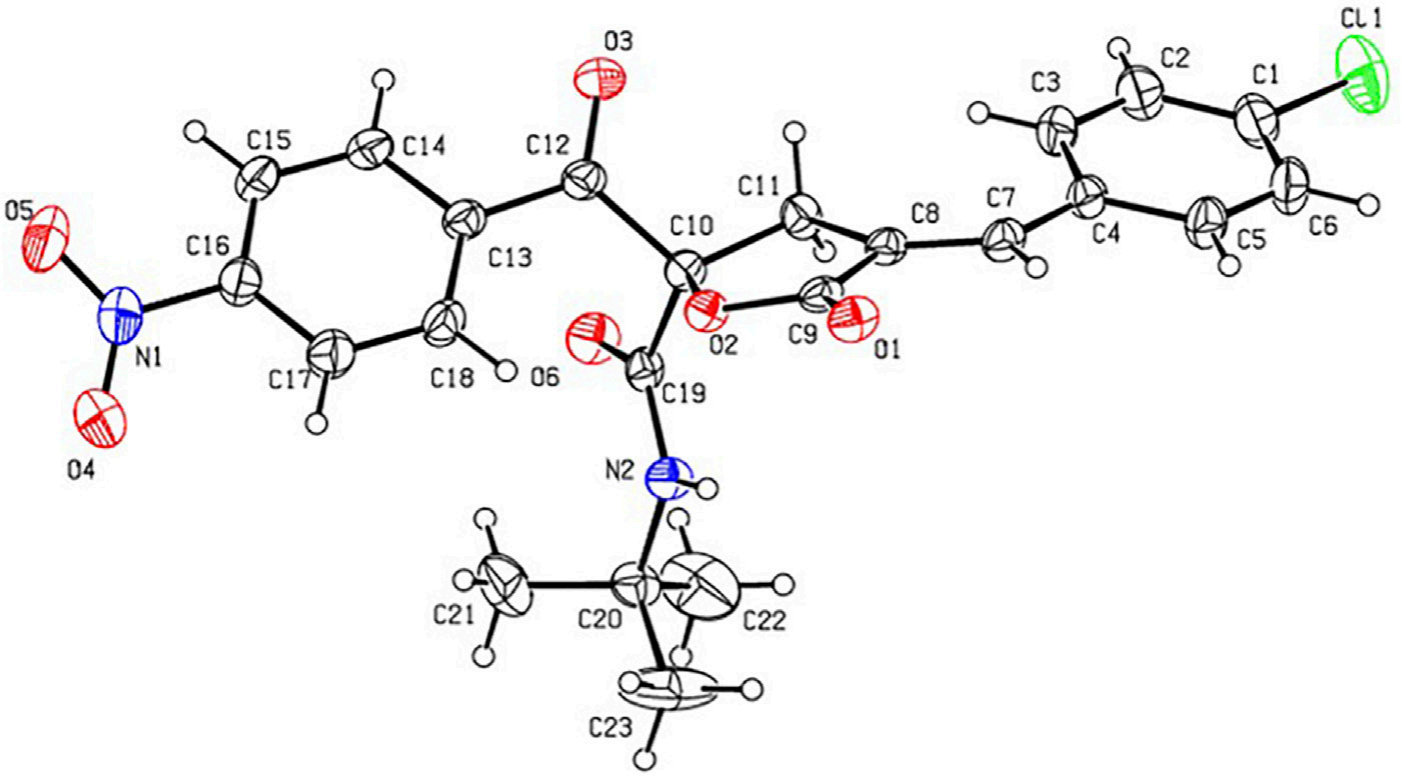
Crystal structure diagram of compound RPDFB (CCDC 2179210). Note: black, carbon; white, hydrogen; red, oxygen; purple, nitrogen; green, chlorine.

**FIGURE 2:**
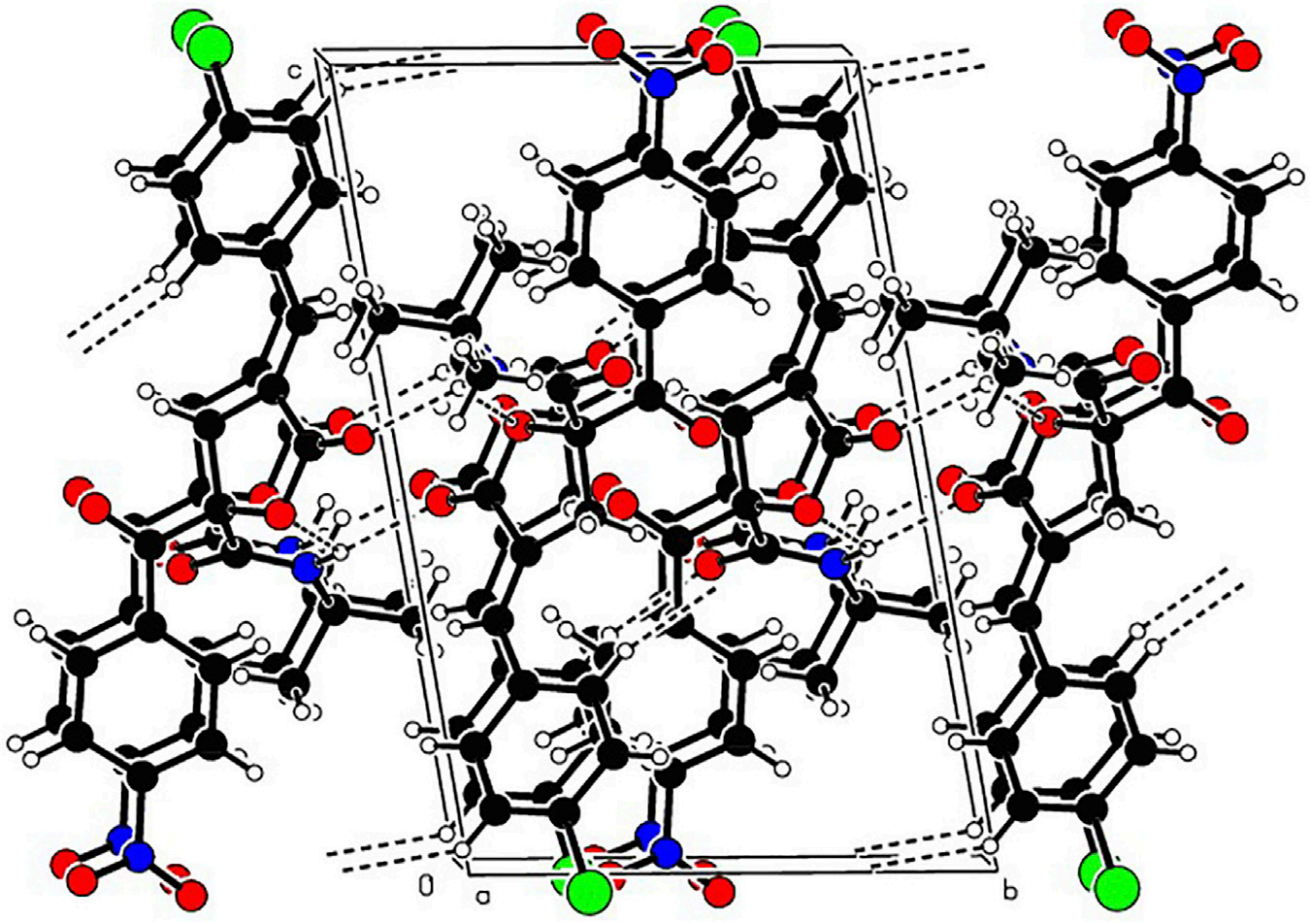
Packing of the crystal structure of compound RPDFB. Note: black, carbon; white, hydrogen; red, oxygen; purple, nitrogen; green, chlorine.

In order to determine the structure of RPDFB, the single crystal of compound RPDFB was cultured. The crystal belongs to triclinic system and the space group were P-1, a = 8.155 (3)Å, b = 9.732 (3)Å, c = 14.809 (5)Å, *α* = 97.446 (5)°, *β* = 98.874 (5)°, *γ* = 101.753 (5)°, *V* = 1,121.1 (6)Å^3^, *Z* = 2, *Dc* = 1.353 Mg/m^3^, *µ*(Mo*Ka*) = 0.212 mm^−1^, F (000) = 476. The crystal structure was solved by the direct method (SHELXL-97), and all the non hydrogen atoms were obtained by multi round Fourier synthesis. The hydrogen atoms were acquired by direct hydrogenation after theoretical calculation. The coordinates of all non hydrogen atoms and heterogeneous thermal parameters were corrected to convergence by the full matrix least square method. The final deviation factor was *R* = 0.0298, *wR* = 0.1444. On the final difference Fourier diagram, the heights of the maximum and minimum electron density peaks were 0.392, −0.392 e/Å^3^, respectively. The single crystal diffraction pattern and the crystal packing pattern of compound RPDFB were showed in Figure 1 and Figure 2, respectively.

### 2.3 Antifungal effect of (E)-2-aroyl-4-arylidene-5-oxotetrahydrofuran derivatives RPDFB, RPDFC, RPDFI, RPDFJ

**FIGURE 3:**
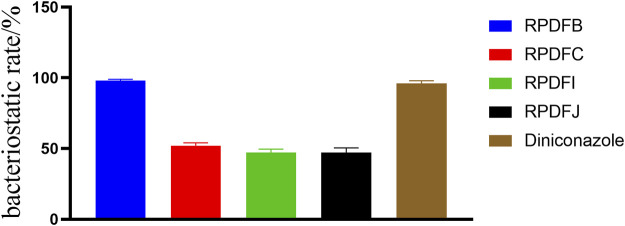
Antifungal activity of (E)-2-aroyl-4-arylidene-5-oxotetrahydrofuran derivatives, against-*Gibberella zeae*.

The growth inhibition activity of the target compounds RPDFB, RPDFC, RPDFI, RPDFJ against *Gibberella zeae* were tested by toxic medium method. The results (Figure 3) showed that at the concentration of 50 μg/ml, the four compounds showed certain inhibitory effects on *Gibberella zeae*, but the inhibitory activities were significantly different. Among them, the inhibition rate of RPDFB was as high as 98%, while the inhibitory effect of other compounds was close to 50%. Under the same control conditions, the growth inhibition rate of diniconazole on *Gibberella zeae* was 96%.

**FIGURE 4:**
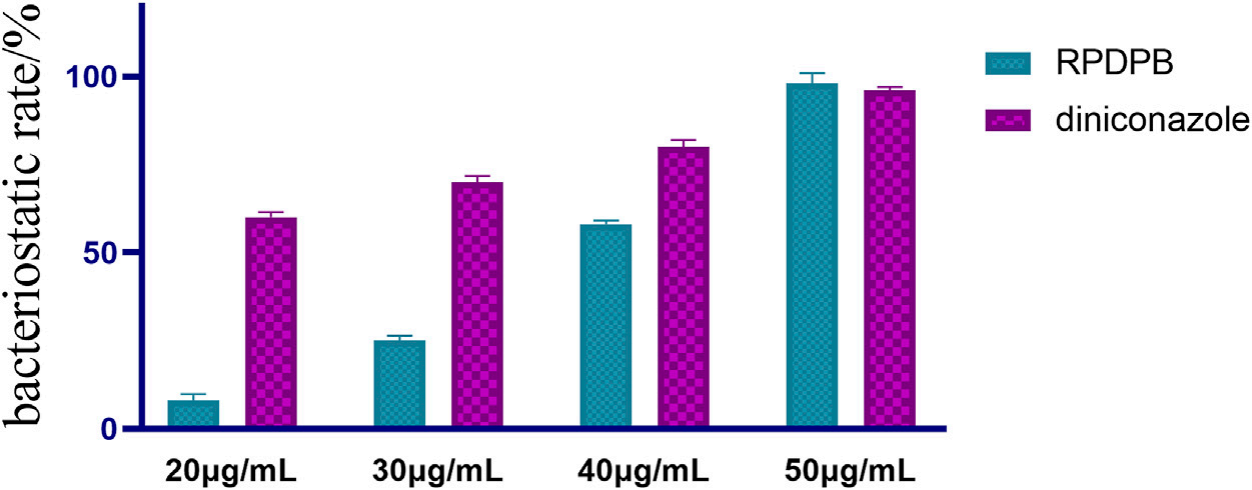
Comparison of inhibitory activities of RPDFB and diniconazole against-*Gibberella zeae*.

Then, the compound RPDFB with better inhibitory activity was selected for concentration gradient control experiment with diniconazole. The results (Figure 4) showed that when the concentration of diniconazole was 30 μg/ml, the growth inhibition rate was as high as 96%, and with the increase of the concentration, the inhibition rate was almost unchanged, even if the concentration decreased to 15 μg/ml, the growth inhibition rate was still up to 90%. When the concentration of RPDFB was 50 μg/ml, the inhibition rate was higher than that of diniconazole. However, when the concentration decreased, the inhibition rate decreased to 58% at 40 μg/ml, and the inhibition activity was not obvious at 30 μg/ml. Our results showed that the compound RPDFB showed a good inhibitory effect on *Gibberella zeae* at 50 μg/ml, and its inhibitory effect was better than that of diniconazole with the concentration more than 50 μg/ml, which is worth further exploration.

## 3 Materials and methods

### 3.1 General information

The melting point was measured by X-4 melting point instrument (uncorrected thermometer) produced by Beijing Rayleigh Analytical Instrument Company. MS was determined by Finnigan-trace-MS analyzer (direct injection method). Elemental analysis was performed by vario-EL-III analyzer. ^1^H NMR and ^13^C NMR were determined by a 600 MHz high resolution nuclear magnetic resonance spectrometer (Varian-Mercury-Plus 600) or 150 MHz high resolution nuclear magnetic resonance spectrometer (Varian-Mercury-Plus 150). The solvent was deuterium chloroform (CDCl_3_) with TMS internal standard. Single crystal diffraction was determined by Bruker-Smart-AXS-CCD X-ray single crystal diffractometer. The reagent used is sinopharm group (or imported) analytical pure.

### 3.2 One-pot synthesis of (E)-2-aroyl-4-arylidene-5-oxotetrahydrofuran derivatives RPDFB, RPDFC, RPDFI, RPDFJ

Dry Baylis-Hillmanic acid (1 mmol) and 2 ml [BMIM][PF_6_] were added to a 25 ml round-bottom flask. After stirring, aryl glyoxals (1 mmol) and isocyanide (1 mmol) were added successively. After shaking, the flask was placed into a microwave reactor with a power of 300 w. Magnetic stirring and interval heating, heating every 8 s, stop 8 s. Repeat several times, and the reaction time is 16 min. Then 2 ml K_2_CO_3_ (0.5 mmol) aqueous solution was divided into 10 parts, and the pH value of the system was adjusted 10 times. Each reaction time is 96 s (heating for 8 s, stopping for 8 s, a total of 6 times). After the reaction was complete (TLC detection), the mixture was chilled overnight and the precipitate was filtered, washed by water, recrystallized from ethanol, then (*E*)-4-arylidene-5-oxotetrahydrofuran derivatives (RPDF serial chemicals) was obtained.

#### 3.2.1 (*E*)-*N*-*tert*-butyl-4-(4-chlorobenzylidene)-2-(4-nitrobenzoyl)-5-oxotetrahydrofuran-2-carboxamide (RPDFB) (Wang et al., 2021)

White crystals (0.35 g, yield 78%), mp 143–144°C; ^1^H NMR (CDCl_3_, 600 MHz) δ (ppm) 8.31 (d, *J* = 8.4 Hz, 2H, Ar-H), 8.22 (d, *J* = 7.8 Hz, 2H, Ar-H), 7.58–7.46 ((m, 5H, Ar-H and = CH), 6.55 (s, 1H, NH), 4.40 (d, *J* = 18.6 Hz, 1H, CH_2_^a^), 3.40 (d, *J* = 18.0 Hz, 1H, CH_2_^b^), 1.36 (s, 9H, 3CH_3_); ^13^C NMR (CDCl_3_, 150 MHz) δ (ppm) 189.2, 169.0, 167.0, 150.5, 138.7, 138.2, 137.1, 132.0, 131.7, 130.6, 129.4,123.6, 120.5, 85.7, 52.6, 29.0, 28.4. MS (*m/z*, %) 456 (M^+^, 4), 356 (66), 306 (95), 250 (100), 150 (66), 57 (74). Anal. Calcd for C_23_H_21_ClN_2_O_6_: C, 60.46; H, 4.63; N, 6.13. Found: C, 60.21; H, 4.68; N, 6.19.

#### 3.2.2 (*E*)-*N*-*tert*-butyl-4-(4-methoxybenzylidene)-2-(4-nitrobenzoyl)-5-oxotetrahydrofuran-2-carboxamide (RPDFC)

White crystals (0.30 g, yield 68%), mp 222–223°C; ^1^H NMR (CDCl_3_, 600 MHz) δ (ppm) 8.30 (d, *J* = 8.4 Hz, 2H, Ar-H), 8.23 (d, J = 9.0 Hz, 2H, Ar-H), 7.57 (s, 1H, =CH), 7.52 (d, *J* = 8.4 Hz, 2H, Ar-H),6.99 (d, *J* = 8.4 Hz, 2H, Ar-H), 6.64 (s, 1H, NH), 4.41 (d, *J* = 18.0 Hz, 1H, CH_2_^a^), 3.88 (s, 3H, CH_3_),3.33 (d, *J* = 18.0 Hz, 1H, CH_2_^b^), 1.36 (s, 9H, 3CH_3_); ^13^C NMR (CDCl_3_, 150 MHz) δ (ppm) 189.4, 169.7,167.3, 161.7, 150.4, 140.0, 138.4, 132.5, 132.5, 131.3, 123.5, 116.7, 114.5, 85.7, 55.6, 52.4, 33.7, 28.4. MS (*m/z*, %) 452 (M^+^, 4), 352 (24), 302 (100), 246 (86), 150 (25), 57 (23). Anal. Calcd for C_24_H_24_N_2_O_7_:C, 63.71; H, 5.35; N, 6.19. Found: C, 63.94; H, 5.38; N, 6.04.

#### 3.2.3 (*E*)-*N*-*tert*-butyl-2-(4-chlorobenzoyl)-4-(4-fluorobenzylidene)-5-oxotetrahydrofuran-2-carboxamide (RPDFI)

White crystals (0.33 g, yield 78%), mp 180–181°C; ^1^H NMR (CDCl_3_, 600 MHz) δ (ppm) 8.04 (d, *J* = 8.4 Hz, 2H, Ar-H), 7.53–7.12 (m, 7H, Ar-H and = CH), 6.69 (s, 1H, NH), 4.39 (d, *J* = 18.0 Hz, 1H, CH_2_^a^), 3.37 (d, *J* = 18.0 Hz, 1H, CH_2_^b^), 1.35 (s, 9H, 3CH_3_); ^13^C NMR (CDCl_3_, 150 MHz) δ (ppm) 189.2,169.4, 167.2, 163.7 (d, *J* = 250 Hz), 140.4, 138.0, 132.4, 131.5, 130.9, 129.9, 128.7, 120.2, 116.2, 86.0,52.2, 33.8, 28.2. MS (*m/z*, %) 429 (M^+^, 3), 329 (47), 290 (51), 234 (56), 139 (100), 57 (26). Anal. Calcd for C_23_H_21_ClFNO_4_: C, 64.26; H, 4.92; N, 3.26. Found: C, 64.16; H, 4.94; N, 3.42.

#### 3.2.4 (*E*)-*N*-*tert*-butyl-4-(4-fluorobenzylidene)-2-(4-nitrobenzoyl)-5-oxotetrahydrofuran-2-carboxamide (RPDFJ)

White crystals (0.36 g, yield 83%), mp 226–227°C; ^1^H NMR (CDCl_3_, 600 MHz) δ (ppm) 8.31 (d, *J* = 8.4 Hz, 2H, Ar-H), 8.23 (d, *J* = 8.4 Hz, 2H, Ar-H), 7.59–7.18 (m, 5H, Ar-H and = CH), 6.61 (s, 1H, NH), 4.41 (d, *J* = 18.6 Hz, 1H, CH_2_^a^), 3.35 (d, *J* = 18.0 Hz, 1H, CH_2_^b^), 1.36 (s, 9H, 3CH_3_); ^13^C NMR (CDCl_3_, 150 MHz) δ (ppm) 189.3, 169.2, 167.0, 163.9 (d, *J* = 250 Hz), 150.4, 138.9, 138.2, 132.5,130.5, 129.9, 123.5, 119.5, 116.3, 85.7, 52.5, 33.6, 28.5. MS (*m/z*, %) 440 (M^+^, 3), 340 (47), 290 (89), 234 (100), 123 (46), 57 (59). Anal. Calcd for C_23_H_21_FN_2_O_6_: C, 62.72; H, 4.81; N, 6.36. Found: C, 62.69; H, 4.93; N, 6.13.

### 3.3 Single crystal structure detection

A crystal with an external size of 0.10 mm^3^ × 0.10 mm^3^ × 0.10 mm^3^ was selected for X-ray single crystal diffraction experiment and placed on a BRUKER SMART APEX-CCD diffractometer for detection. MoKα rays (wavelength *λ* = 0.71073Å) monochromatized by a graphite monochromator are used as incident radiation. In the range 2.00<θ < 26.55°, a total of 8,168 reflection points were collected by ω-2θ scanning at 298 (2) K, of which 5,150 were independent observable reflections [I ≥ 2σ(I)], R (int) = 0.0247. All the intensity data were corrected by SADABS software.

### 3.4 Antifungal assay

The antifungal activity of the compound RPDFB, RPDFC, RPDFI, RPDFJ at 50 μg/ml was determined against *Gibberella Zeae* by the poison plate technique. The medium was prepared by dissolving 43 g of potato dextrose agar powder (PAD. BR, Solarbio) in 1 L of distilled water and sterilized in autoclave at 125°C for 0.5 h. Next, the 2.0 mg drug was dissolved with 0.2 ml DMSO (AR, Sinopharm) and emulsified with a drop of tween-80 (AR, Sinopharm), then diluted to 2.0 ml with distilled water to obtain intermediate solutions of 1 mg/ml. Add 500 μL intermediate solution, while hot, to a Petri dish containing 9.5 ml PDA medium. After evenly mixing, a working solutions with a concentration of 50 μg/ml is obtained. As in the above method, enazole and enazolone were added as positive control group, and no drug was added as blank control group. Then a blank PDA with a diameter of about 5 mm containing *Gibberella Zeae* was selected by the inoculation ring, and the mycelium was placed in the middle of the PDA containing the above-mentioned drugs. The lid of the Petri dish was immediately covered. The Petri dish was placed in a constant temperature incubator and cultured at 25°C for 3 days. The inhibition effect on the growth of *Gibberella Zeae* was observed and the diameter of plaque was measured. Three parallel experiments were performed. Inhibition rate (%) = (control colony diameter - treatment colony diameter)/(control colony diameter - initial plaque diameter).

## 4 Conclusion

We improved the synthesis method of (*E*)-*N*-*tert*-butyl-4-(4-chlorobenzylidene)-2-(4-nitrobenzoyl)-5-oxotetrahydrofuran-2-carboxamide (RPDFB) by using ionic liquid as medium and microwave assisted method, which made the synthesis process more green and efficient. Meanwhile, three new compounds were synthesized by this method, and the chemical structures of four compounds were verified by spectroscopic method. The single crystal structure of RPDFB was also cultured and analyzed. Moreover, all the four compounds had inhibitory effects on *Gibberella Zeae*, and RPDFB had better anti-fungal effect. However, compared with enazole, the antibacterial effect of RPDFB still lags behind, which is worthy of further structural optimization. In conclusion, we synthesized a series of γ-lactone derivatives with a new synthesis strategy that is more environmentally friendly and efficient, hoping to provide a little help for the “greening” of organic synthesis.

## Data Availability

The datasets presented in this study can be found in online repositories. The names of the repository/repositories and accession number(s) can be found in the article/Supplementary Material.
