# Supplementary material for: Green synthesis, crystal structure, and antifungal activities of (E)-4-arylidene-5-oxotetrahydrofuran
Source: Front Chem. 2022 Sep 9;10:997095. doi: 10.3389/fchem.2022.997095 (PMC9500531; doi:10.3389/fchem.2022.997095)
Supplement: Supplementary file 1 [file DataSheet1.docx]

RPDFB ^1^H NMR

RPDFB ^13^C NMR

RPDFC ^1^H NMR

RPDFC ^13^C NMR

RPDFI ^1^H NMR

RPDFI ^3^C NMR

RPDFJ ^1^H NMR

RPDFJ ^3^C NMR
